# Microplastics in Sediments of East Surabaya, Indonesia: Regional Characteristics and Potential Risks

**DOI:** 10.3390/ijerph191912348

**Published:** 2022-09-28

**Authors:** Achmad Chusnun Ni’am, Fahir Hassan, Ruei-Feng Shiu, Jheng-Jie Jiang

**Affiliations:** 1 Department of Environmental Engineering, Institut Teknologi Adhi Tama Surabaya, Jalan Arief Rahman Hakim, Surabaya 60117, Indonesia; 2 Department of Civil Engineering, Chung Yuan Christian University, Taoyuan 320314, Taiwan; 3 Advanced Environmental Ultra Research Laboratory (ADVENTURE), Department of Environmental Engineering, Chung Yuan Christian University, Taoyuan 320314, Taiwan; 4 Institute of Marine Environment and Ecology, National Taiwan Ocean University, Keelung 20224, Taiwan; 5 Center for Environmental Risk Management (CERM), Chung Yuan Christian University, Taoyuan 320314, Taiwan

**Keywords:** microplastics, risk assessment, source apportionment, pollution characteristics

## Abstract

The presence of microplastics (MPs) in marine environments has become increasingly apparent. Owing to the lack of effective solid waste management, Indonesia is the second largest producer of ocean plastic waste after China. Currently, information about pollution of MPs in the sediments of East Surabaya, Indonesia, is not available, and this issue is addressed in this study for the first time. Sediment samples were collected from 16 sampling sites along urban and mangrove coastal areas. MPs were observed in most of the sampling sites, with abundances ranging from ND (not detected) to 598 items/kg. MP shapes constituted fragments (30%), foam (28%), granules (22%), and fibers (20%). The 500–1000 µm fraction was the dominant size of MPs. Polypropylene was the major polymer constituent, followed by high-density polyethylene and polyethylene. Findings from Spearman’s correlation coefficients, principal component analysis, and hierarchical cluster analysis reveal that the spatial pattern of MPs is closely related to coastal characteristics and population density. MPs in different coastal regions were assessed by the polymer risk index. Results reveal that coastal areas in the Bulak district exhibit the highest risk. Our results confirm the prevalence of MPs as anthropogenic pollutants in East Surabaya and highlight the importance of management action and education on environmental protection for the mitigation of MP pollution.

## 1. Introduction

With the rapid increase in the disposal of plastics into the marine environment annually (from 4.8 to 12.7 million), the oceans may contain more plastic than fish by 2050 [1]. Microplastics (MPs), plastics with a size of <5 mm, have been observed in significant quantities in the environment. There are increasing concerns about their impact on ecosystems [2]. In the environment, plastic waste can be degraded by physical and chemical processes such as wave action and exposure to ultraviolet light [3]. In the past decade, an increasing number of studies have reported the detection of MPs in marine and terrestrial environments. Considered contaminants of emerging concern, MPs are ubiquitous in the aquatic environment. Thus far, the contamination of MPs in tap water, river water, seawater, wastewater, beaches, sediments, and organisms has been reported [4,5,6,7,8]. 

Based on their polymer types, MPs have different densities. It is estimated that 99% of all plastics entering the ocean will eventually settle in sediments [9]. Therefore, sediments are expected to be the ultimate sink for MPs. Thus far, several studies have reported the pollution of MPs in Indonesian surface water and sediments [10,11,12,13,14]. However, few studies focus on the occurrence and risk of MPs in sediments of East Surabaya, East Java, Indonesia.

MP abundance is thought to be associated with population density. The higher the population density in an area, the higher the abundance of MPs [15,16,17]. Indonesia is estimated to have a population of 187 million residing within 50 km of the coast. In 2020, the population of Surabaya, which is the largest city in East Java of Indonesia, was 3.1 million. The Surabaya coastal area serves as a center for marine transportation, marine aquaculture, tourism, fisherman residence, and river discharge [13]. In addition, extremely intense human activities occur on the east coast of Surabaya; hence, it is subjected to anthropogenic waste including MPs from terrestrial sources. Owing to the excessive generation of improperly managed waste (3.22 million tons per year), an estimated 0.48–1.29 million metric tons of plastic are discharged into the ocean annually, leading to considerable pollution in Indonesia [18]. Therefore, it is crucial to establish the characteristics and distribution of MP pollution and its key sources in Indonesia. For this purpose, this study investigated the occurrence and characteristics of MPs as well as their pollution topography in East Java. Principal component analysis (PCA) and hierarchical cluster analysis (HCA) were simultaneously performed to gain a better understanding of the long-term anthropogenic contribution of this highly populated area.

## 2. Materials and Methods

### 2.1. Study Area and Sample Collection

**Figure 1:**
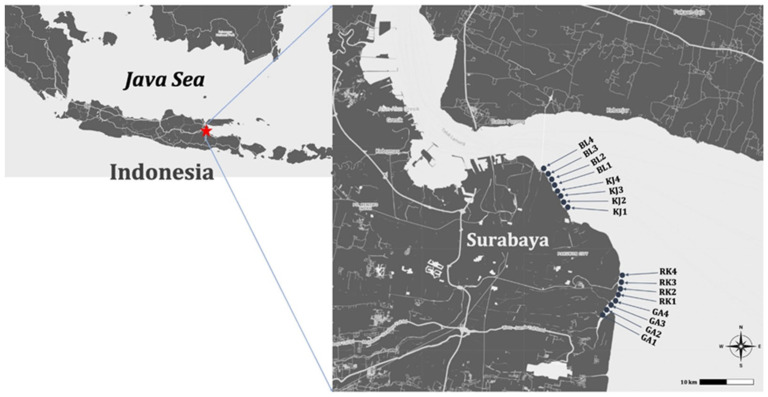
Study area and sampling locations.

Based on the geomorphology and population density, two main coastal areas were examined in this study: mangrove (Rungkut District and Gunung Anyar District) and urban (Bulak District and Kenjeran District) coastal areas. In August 2017, 16 sites were sampled for surface sediments. Of these, RK1, RK2, RK3, RK4, GA1, GA2, GA3, and GA4 represented the mangrove coastal areas, while BL1, BL2, BL3, BL4, KJ1, KJ2, KJ3, and KJ4 represented the urban coastal areas (Figure 1). The coastal areas of the Rungkut District and Gunung Anyar District are protected marine mangrove forests in East Java. The coastal areas of the Bulak District and Kenjeran District are muddy beaches that directly face the Madura Strait, and land use is dominated by tourism and fisherman residence. 

The 16 sites were sampled for surface sediments in triplicate. An Ekman grab sampler was used to obtain samples of the surface sediment (depth 0–2 cm). Next, a stainless-steel spatula was used to transfer the samples into aluminum foil sampling bags, which were then stored in an incubator filled with ice cubes. During sample collection and storage, only glass, stainless steel, and aluminum instruments were used to avoid contamination. 

### 2.2. Extraction and Identification of Microplastics

MPs were extracted according to methods reported previously with some modifications [19,20]. Briefly, approximately 300 g of the sediment sample was weighed, dried in an oven, and weighed again in a glass beaker. After the addition of 250 mL of a saturated NaCl solution, the samples were stirred for 10 min to ensure that the MPs were suspended. To remove NaCl, the supernatant liquid was filtered through a 25 µm steel sieve using a vacuum suction device after 12 h of settlement. To remove organic matter, 30% H_2_O_2_ was added to a glass beaker, and the material was suspended in a saturated NaCl solution. After the supernatant liquid was filtered through a 25 µm steel sieve, it was washed at least five times with distilled water to remove NaCl. The MP specimens were transferred from the steel sieve to Petri dishes for further MP examination. 

MPs were identified and counted under a stereoscopic microscope (Olympus, IX83, Tokyo, Japan) equipped with a digital camera. The MPs were categorized into four groups according to their shape composition: foam, fragments, granules, and fibers. MPs were further classified into four groups according to their size: <100 µm, 100–500 µm, 500–1000 µm, and 1000–5000 µm. Micro Fourier transform infrared (µ-FTIR) spectroscopy was employed to analyze all particles (Shimadzu, AIM-9000, Kyoto, Japan) in the attenuated total reflectance (ATR) mode. Each scan was accumulated as an average of 64 scans in the spectral range 4000–700 cm^−1^ at a resolution of 0.25 cm^−1^. For each particle, three replicates of the ATR-FTIR spectrum were recorded. Each spectrum was directly compared with the library of polymers provided by Shimadzu to verify the polymer type. The matching spectrum with a quality index of ≥70% was accepted.

### 2.3. Quality Assurance and Quality Control

Nitrile gloves and non-textile laboratory coats were worn during the experiments. Experimental areas were kept clean to prevent contamination. Anhydrous ethanol and ultrapure water were used to rinse all glass vessels three times, and aluminum foil was subsequently used to cover them when not in use. A culture dish with filter paper was used in the experimental area to examine airborne MPs. The results indicate that such MPs were not present. To determine the effect of other factors, a blank experiment was conducted with ultrapure water. MPs were not detected in the blank samples. Hence, experimental contamination was insignificant.

### 2.4. Statistical Analysis

In this study, the data set was subjected to PCA and HCA for data exploration and description. PCA is an explorative tool to extract the number of components needed to explain the observed data variance. HCA is a statistical method used to classify samples into clusters according to their similarity and various cluster rules. To simplify the analysis, components explaining only a small margin of variance (<5%) were avoided and assumed to be mostly caused by background noise and noise sources. Varimax was used for rotation in PCA, and only the highest coefficients were retained in the varimax normalized matrix. Ward’s hierarchical agglomerative method of clustering and Euclidean distance measure was used to analyze the relationships between polymer types.

### 2.5. Risk Assessment

Currently, most studies on MP risks focus on their harm and additive effects, but only a few analytical models are available to assess the MP risks posed to human health. Based on this, risk index (*H*) values for MPs were used to preliminarily assess the risks of MPs in sediments in this study by referring to the study reported by Li, et al. [21]. Hazard scores and polymer types were used as indicators to assess the risks associated with MPs:

$$H=\sum P_{n}\times S_{n}$$

where *H* is the calculated polymer risk index, *P_n_* is the percentage of each MP polymer type calculated from the ratio of MP concentrations for each polymer type to the total MP concentration, and *S_n_* is the hazard score of the MP polymer, which has been reported previously by Lithner, et al. [22]. 

## 3. Results and Discussion

### 3.1. Occurrence of MPs

**Figure 2:**
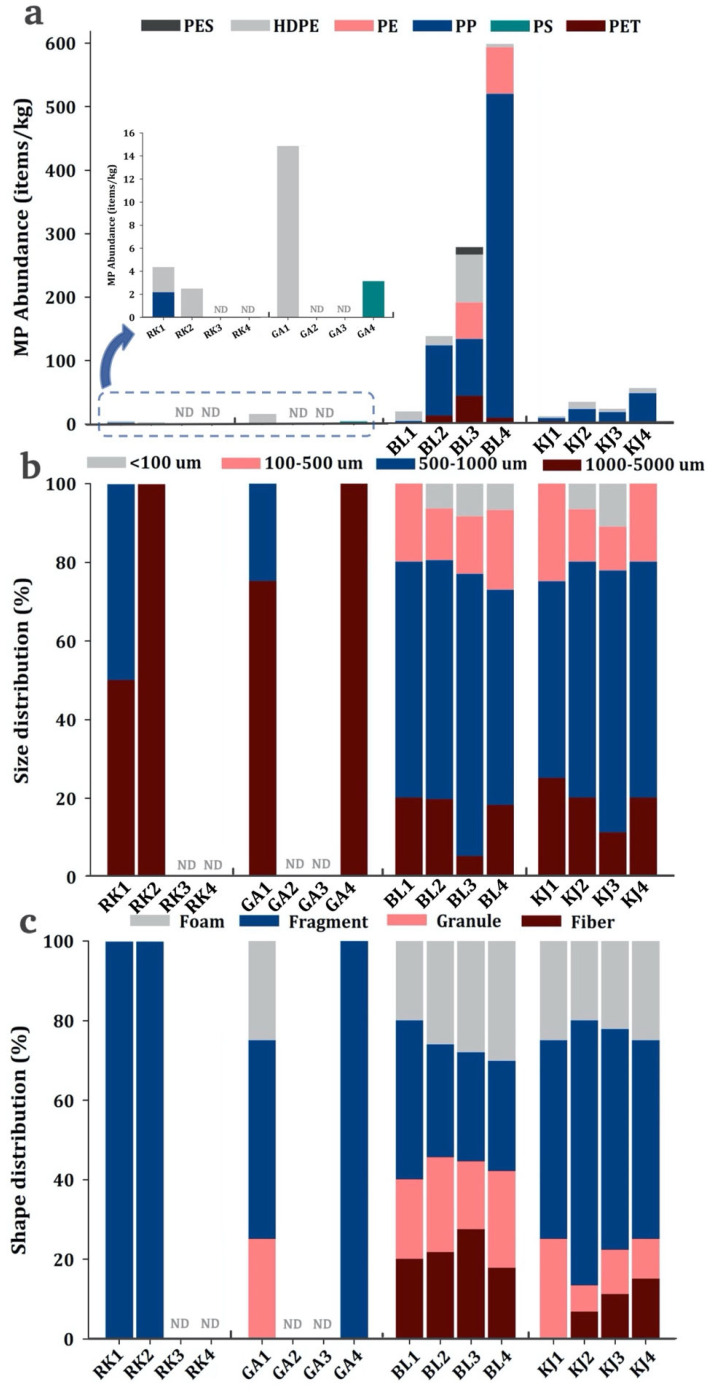
Distribution of MP (**a**) compositions, (**b**) sizes, and (**c**) shapes at sampling stations.

MPs were found in all sediment samples except for four samples from the mangrove coastal area (i.e., RK3, RK4, GA2, and GA3). The abundance of MPs in surface sediments ranged from ND (not detected) to 598 items/kg, with higher abundance closer to urban areas (BL1–BL4 and KJ1–KJ4) and lower abundance in the mangrove areas (RK1–RK4 and GA1–GA4) (Figure 2). The average abundance of MPs in the surface sediments was 73.9 ± 157 items/kg dry weight (dw), which is similar to those reported in the Jagir Estuary, Bohai Sea, Yellow Sea, and South China Sea [11,20,23]. The abundance of MPs in sediments from the urban coastal areas (145 ± 204 items/kg) was considerably greater than that in the mangrove coastal areas (3.11 ± 5.06 items/kg). The higher MP abundance closer to urban areas may be attributed to terrestrial runoff and mariculture activities. 

The MP abundance in the surface sediments at BL4 was 40 times greater than that at GA1 and RK1, which may be attributed to the dense population in the surrounding area. This result is in agreement with that of a previous study, which reported that MP abundance is significantly correlated with population density and anthropogenic activities [24]. In addition, such high MP pollution at BL1–BL4 may be attributed to the high generation rate of municipal solid waste in Surabaya. The inadequate solid waste management infrastructure and services have led to a greater amount of improperly disposed municipal solid waste. 

Despite this background information, data on MP abundance in Indonesia are limited. Thus far, only a few studies have focused on different regions and seas in Indonesia [10,11,12,14,25,26]. Data from these studies were compared with the results obtained in this study. The MP concentrations reported here are considerably lower than those reported for the coastal sediments of Jakarta Bay (18,405–38,790 items/kg), but MP concentrations on the East Surabaya coast are comparable with those reported in Sumatera, Jagir Estuary, and Banten Bay [11,12,26].

### 3.2. Characteristics of MPs

As illustrated in Figure 2, most of the MP sizes range between 500 µm and 1000 µm (63%), while the MP percentages within the size categories <100 µm, 100–500 µm, and 1000–5000 µm are 7%, 12%, and 18%, respectively. In terms of spatial distribution, MP sizes between 500 µm and 1000 µm were more dominant in the urban and mangrove coastal areas, and MP sizes of <500 µm were not observed in the mangrove coastal areas.

In the study areas, fragments were found to be the main shape of MPs (30%), followed by foam (28%), granules (22%), and fibers (20%). The MP distribution differed in each area. The fragment component of the sediment samples collected from the mangrove coastal areas (RK1, RK2, GA1, and GA4) was greater (50–100%) than that from the urban coastal areas (27–66%). Most fragment shapes possibly originated from the breakdown of large plastic goods via mechanical and ultraviolet degradation in the environment. In addition, food packaging materials, containers, toys, and other items may be important sources of fragments. [27]. Foam-shaped MPs were possibly derived from food-packaging materials, whereas granule-shaped MPs possibly originated from plastic raw materials or personal care products [28]. Fiber-shaped MPs possibly originated from textile materials, clothes, rope, fishing gear, and fishery activities, indicative of domestic waste contribution [29]. 

### 3.3. Composition Patterns and Potential Sources of MPs

**Figure 3:**
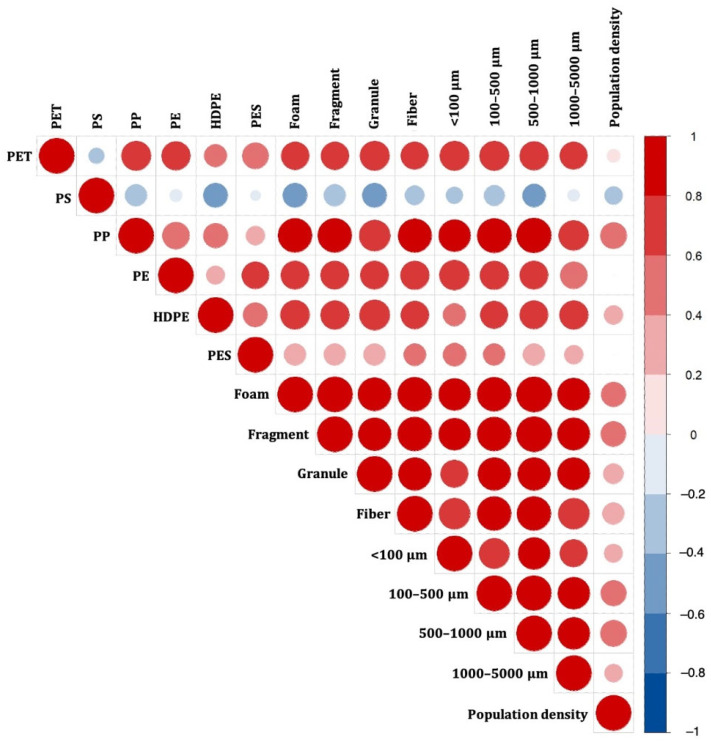
Spearman’s rank correlation coefficient between MP characteristics and population density.

Based on the µ-FTIR analysis results, six components of MPs were identified in the sediments of East Surabaya: polyester (PES, 1.27 g/cm^3^), high-density polyethylene (HDPE, 0.96 g/cm^3^), poly-ethylene (PE, 0.93 g/cm^3^), polypropylene (PP, 0.905 g/cm^3^), polystyrene (PS, 1.05 g/cm^3^), and polyethylene terephthalate (PET, 1.29 g/cm^3^). PP was the dominant component, accounting for 70.1%, followed by HDPE (11.9%), PE (11.5%), PET (5.27%), PES (0.98%), and PS (0.20%) (Figure 2). A relatively high abundance of PP and PE was detected in the sediments collected from the urban coastal areas, especially in BL3 and BL4. PET and PES were only found in the sediments collected from the urban coastal area, while relatively minor proportions of HDPE and PS were found in the sediments collected from the mangrove coastal areas (RK1, RK2, GA1, and GA4). Generally, PP, HDPE, and PE were the major polymers found in the sediments collected from East Surabaya; this result is consistent with that reported by Lestari, et al. [30], in which PP, PE, and LDPE were the major MP compositions found in the surface water in Surabaya River. Biofouling and biological ingestion–excretion adsorbed onto MPs can increase MP density; hence, MPs may settle on surface sediments despite the fact that PP and PE are less dense than seawater [31,32]. Population density analysis provides more specific information regarding potential sources, which is typically applied as a predictor variable of MP pollution. In this study, MP pollution in sediments was closely related to the dominant MP characteristics (PP, foams and fragments, and 100–500 µm and 500–1000 µm) and population density (Figure 3).

**Figure 4:**
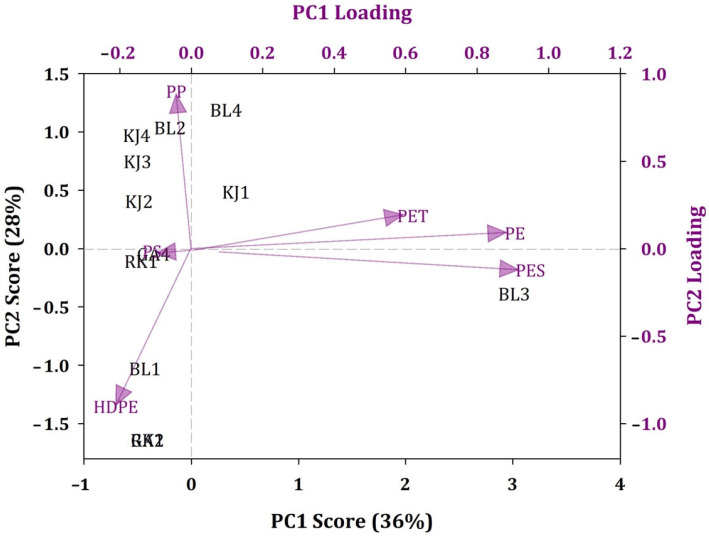
Principal component analysis score and loading plots of the distribution of MPs in the sediments of East Surabaya, Indonesia.

PCA identified two principal components, PC1 and PC2, accounting for 36% and 28% of the total variance, respectively. Figure 4 shows the PCA score and loading plots of the MP distribution of sediments. PC1 exhibits strong contributions from PE, PES, and PET, and PC2 is dominated by high positive loadings of PP and high negative loadings of HDPE. Notably, the urban coastal areas of the Bulak District and Kenjeran District, which contained more PP, are grouped together, while the mangrove coastal areas of the Rungkut District and Gunung Anyar District are grouped together based on the dominant component. This is indicative of distinct origins.

**Figure 5:**
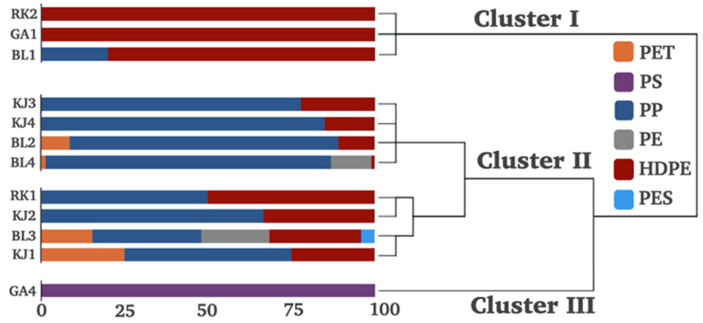
Hierarchical cluster analysis and compositional profiles of MPs in the sediments of East Surabaya, Indonesia.

Comprehensive data analysis was conducted on the urban and mangrove coastal areas in East Surabaya. Figure 5 shows fingerprinting and a dendrogram of the sampling sites obtained from HCA. Three well-differentiated clusters can be seen: (I) a cluster containing sampling sites with high compositional fractions of HDPE in RK2, GA1, and BL1; (II) a cluster formed by a majority of the urban coastal sampling sites, characterized by relatively high compositional fractions of PP; (III) a single sampling site from a mangrove coastal area with a high compositional fraction of PS. Of the three clusters, Cluster II was the largest, constituting most of the sampling sites in the urban coastal area (BL2–BL4 and KJ1–KJ4) and station RK1, while Cluster I and Cluster III contained most of the sampling sites in the mangrove coastal area. 

### 3.4. Risk Assessment of MPs

**Figure 6:**
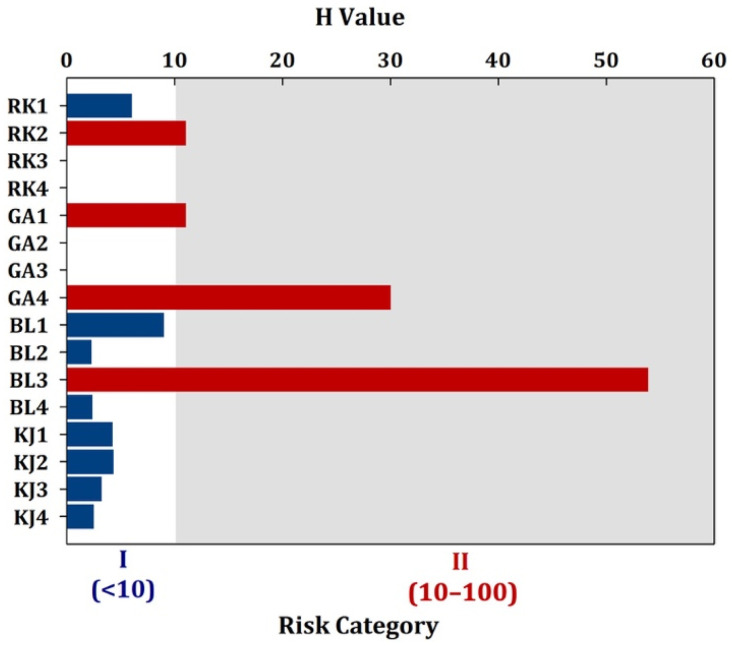
Environmental risk evaluation of MPs in the sediments of East Surabaya, Indonesia.

Several studies have reported the toxic effects of MPs on organisms, but few studies have evaluated these effects in a systematic way for a particular area [27,33]. In this regard, the MP risk index (*H*) values for each sampling site were calculated and classified according to the method outlined above. MPs with *H* values of 10, 10–100, 100–1000, and >1000 were categorized into risk groups I, II, III, and IV, respectively. Risk category IV indicates the highest risk. As shown in Figure 6, the risk categories of RK1, BL1, BL2, BL4, KJ1, KJ2, KJ3, and KJ4 were of risk category I, indicative of relatively low risk in this area. RK2, GA1, GA4, and BL3 represented risk category II, corresponding to a higher risk. Based on our results, the higher risk was not highly related to urban coastal sampling sites, indicating a weak relationship between risks and human population. In general, PP had a relatively small risk compared with PET, PS, PE, and HDPE, while PES had a greater risk. It is likely that the high risk levels of BL3 are related to the presence of PES, which deserves more attention. In contrast, the *H* values for MPs in most sites were less than 10, indicative of low risk. Hence, it is crucial to refining the model further by quantifying human exposure to MPs and gathering risk data on the combined effects of MPs and other pollutants. Additional data are needed to clarify the future threat posed by MPs to humans.

## 4. Conclusions

In this study, sediment samples collected from eight urban coastal areas and eight mangrove coastal areas contained microplastics (MPs) with varying abundance. The abundance of MPs along the coast near densely populated areas was one to two orders of magnitude greater than that along the coast close to protected marine mangrove forests. Characteristics of MPs found in urban coastal areas were different from those found in mangrove coastal areas. The majority of MPs found in the mangrove coastal areas were characterized as fragments with a large size (500–5000 µm) and were composed of high-density polyethylene (HDPE), while MPs found in urban coastal areas were mainly characterized as fragments, foam, and fibers with a small size (<100–500 µm) and composed of polypropylene (PP). A significant relationship was observed between population density and the dominant MP characteristics (PP, foam and fragments, 100–500 µm and 500–1000 µm). The long-term and large-scale monitoring of MPs in different types of coastal areas should be considered in further global schemes, which are crucial for understanding the fate of MPs during the journey from land to sea.

## Data Availability

Data will be available upon reasonable request to the corresponding author.
